# Methyltransferases from RiPP pathways: shaping the landscape of natural product chemistry

**DOI:** 10.3762/bjoc.20.147

**Published:** 2024-07-18

**Authors:** Maria-Paula Schröder, Isabel P-M Pfeiffer, Silja Mordhorst

**Affiliations:** 1 Pharmaceutical Institute, Department of Pharmaceutical Biology, University of Tübingen, Auf der Morgenstelle 8, 72076 Tübingen, Germany

**Keywords:** biocatalysis, methylation, post-translational modifications, ribosomal peptides, SAM-dependent enzymes

## Abstract

This review article aims to highlight the role of methyltransferases within the context of ribosomally synthesised and post-translationally modified peptide (RiPP) natural products. Methyltransferases play a pivotal role in the biosynthesis of diverse natural products with unique chemical structures and bioactivities. They are highly chemo-, regio-, and stereoselective allowing methylation at various positions. The different possible acceptor regions in ribosomally synthesised peptides are described in this article. Furthermore, we will discuss the potential application of these methyltransferases as powerful biocatalytic tools in the synthesis of modified peptides and other bioactive compounds. By providing an overview of the various methylation options available, this review is intended to emphasise the biocatalytic potential of RiPP methyltransferases and their impact on the field of natural product chemistry.

## Introduction

In the complex landscape of natural product biosynthesis, ribosomally synthesised and post-translationally modified peptides (RiPPs) stand out as a fascinating class of compounds with both structural diversity and unique bioactivities [1]. RiPP natural products are typically encoded in a biosynthetic gene cluster (BGC) and produced following defined biosynthetic rules. Initially, a precursor peptide encompassing a core peptide, a leader part and/or a follower part, is synthesised at the ribosome. Next, distinct maturases encoded in the same BGC as the precursor peptide install post-translational modifications in the core peptide. Finally, a protease releases the modified core peptide, creating the mature natural product [2].

The transfer of a methyl group is a common post-translational modification in RiPP biosynthesis; this reaction is catalysed by *S*-adenosylmethionine (SAM or AdoMet)-dependent methyltransferases (MTs). These enzymes are known for their excellent chemo-, regio-, and stereoselectivity [3–6]. Their ability to introduce methyl groups at specific positions within ribosomal peptides equips the resulting natural products with a spectrum of chemical complexity, contributing to the broad range of biological activities exhibited by RiPPs.

Bioactivities attributed to RiPPs include a wide range of effects, such as antibiotic, antifungal, antiviral, antiparasitic, antitumour, analgesic, anti-inflammatory, antidiabetic, antihypertensive, and anti-parkinsonian [7–8]. This diverse profile makes RiPPs particularly valuable for various therapeutic and medicinal applications. Generally, peptide natural products are promising drug development candidates due to their intermediate molecular weight, which falls between small molecules and large biologicals such as antibodies. Small molecules typically weigh less than 900 Dalton (Da), while the molecular weight of large biologicals ranges from approximately 5,000 to 150,000 Da. Although some small RiPPs may be classified as small molecules, the majority of RiPPs have a molecular weight between 1,000 and 5,000 Da. Peptide natural products exhibit high specificity and binding affinity to their corresponding targets [9–10]. The inhibition of protein–protein interactions is an emerging strategy in the development of novel therapeutics [11]. Binding to proteins allows peptides to disturb protein–protein interactions, which is a challenging task for small molecules.

The principles of RiPP biosynthesis make the RiPP technology an ideal platform for the generation of designed peptides and allow tailored derivatisation of these peptides. The RiPP technology can also be employed to mimic non-ribosomal peptides [12–13], since it is substantially more challenging to engineer non-ribosomal peptide synthetase (NRPS) pathways. Non-ribosomal peptides are often produced by giant multi-modular enzyme complexes (type I NRPS) and they are most commonly involved in the specialised metabolism of bacteria and fungi. Each NRPS module is minimally composed of a condensation (C) domain, an adenylation (A) domain, and a peptidyl carrier protein (PCP or thiolation/T domain), and one module typically incorporates one amino acid into the final natural product. In contrast to ribosomal peptide biosynthesis, many non-proteinogenic amino acids can be incorporated as building blocks. However, RiPP pathways are more flexible and they represent interesting biotechnological production routes.

In this review, we will focus on post-translational methylation reactions. A single methyl group has the ability to modulate a drug’s efficacy by orders of magnitude; this occurrence is also called the ‘magic methyl effect’ [14–15]. Compared to chemical methylation reactions, a biocatalytic methylation often offers several advantages. MTs exhibit high selectivity and are environmentally friendly, whereas chemical methylating agents are usually toxic and unselective [3]. Nonetheless, a drawback is that MTs rely on the costly cofactor SAM, which is required in stoichiometric amounts. Furthermore, it is unstable in physiological conditions and produces *S*-adenosylhomocysteine (SAH) as a co-product, which strongly inhibits various MTs [16–19]. To overcome these limitations, numerous in situ SAM supply and SAM regeneration systems have been developed [4,20–26], thereby enabling preparative biocatalytic scale methylation reactions [27]. Such regeneration systems have been widely applied to small molecules [28–30], as well as polyketide natural products [31], and RNA [32]. To the best of our knowledge, these systems have not yet been applied to peptide natural products.

**Figure 1:**
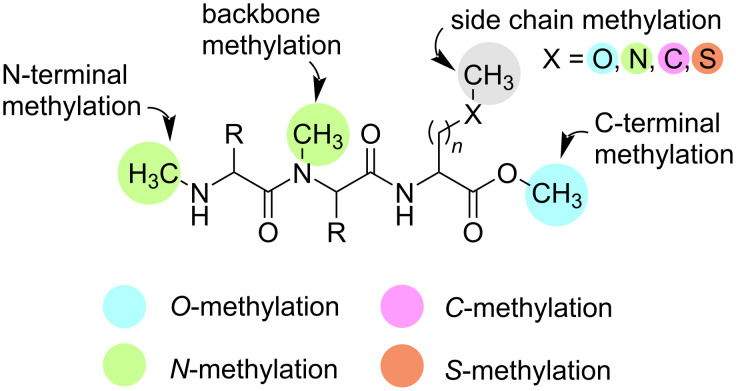
Schematic representation of the different acceptor regions for the methylation of RiPPs discussed in this article.

By providing a comprehensive overview of the various methylation options available within RiPP biosynthetic pathways, this review aims to emphasise the biocatalytic potential of RiPP MTs and their transformative impact on the field of natural product chemistry. Instead of providing an exhaustive list of all identified RiPP MTs to date, we will focus on selected examples for varying acceptor regions to demonstrate the broad range of methylated positions in ribosomally synthesised peptides (Figure 1). This article also highlights the potential application of RiPP MTs in biocatalysis and their ability to enable the production of bioactive compounds with tailored chemical functionalities.

## Review

### Methylation strategies

The main section of this paper discusses different RiPP MTs involved in the biosynthesis of methylated peptide natural products. This section serves as an introduction and outlines additional synthetic strategies for methylated amino acids and peptides. There are three main routes for the synthesis of methylated peptides: chemical synthesis [33–34], in vitro ribosomal synthesis [35], and enzymatic synthesis.

**Figure 2:**
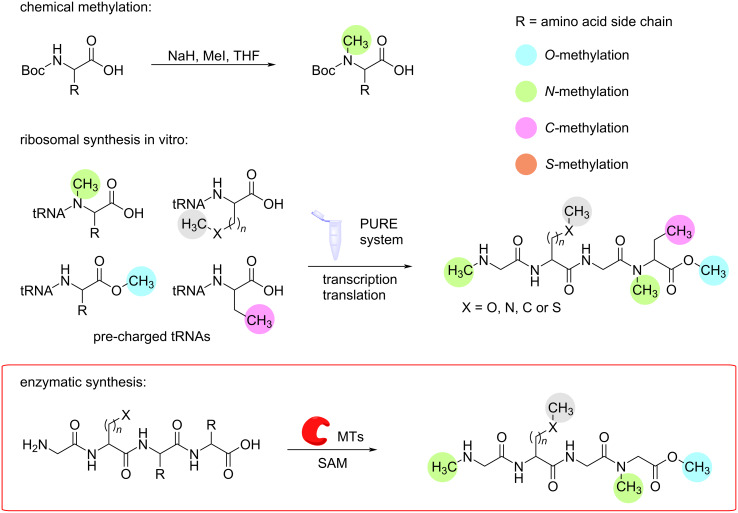
Schematic overview of different methylation strategies for amino acids and peptides. There are several options for chemical methylation (see text), but only one example is shown. Enzymatic synthesis is highlighted in red as this is the focus of this highlight. PURE = protein synthesis using recombinant elements, MT = methyltransferase.

**Chemical synthesis**. Three main categories of reactions are commonly used: reductive amination, reductive ring openings, and the use of methylating agents [34]. In reductive amination, the substrate is usually an aldehyde or amine. After the formation of the iminium ion, it is reduced with the appropriate reagent to form the *N*-methylated amino acid. Different methods have been established using for example benzaldehyde as a protection group, sodium cyanoborohydride as a mild reducing agent, and paraformaldehyde as a methylating agent [36]. Methanol can be used as the methylating reagent in other methods. Here, a palladium on carbon (Pd/C) catalyst processes the dehydrogenation of the alcohol to form the corresponding aldehyde. The subsequently formed imine intermediate is then reduced with H_2_ [37]. When synthesising via a reductive ring opening, typically, an *N*-Fmoc-protected amino acid is condensed with formaldehyde in the presence of *p*-toluic acid in refluxing toluene to yield the 5-oxazolidinone. Reductive ring opening can be achieved by using an excess of triethylsilane in TFA-CH_3_Cl, resulting in the *N*-methylated amino acid as the final product [38]. The third method for chemical *N*-methylation involves the use of protection groups that also enhance the reactivity of the primary amine (Figure 2). Once the amine is deprotonated, an electrophilic methylation reagent donates its methyl group to yield the mono-*N*-methylated amino acid. Common reagents for this reaction include iodomethane (MeI), cyanamide (CH_2_N_2_), and dimethyl sulphate ((CH_3_O)_2_SO_2_). A similar logic can be followed to methylate the C_α_-carbon, with reaction conditions ranging from mild to very harsh. Additionally, stereoselectivity can be achieved by using potassium bis(trimethylsilyl)amide for deprotonation and methylation with MeI [33,39].

Any of these routes would yield methylated amino acids that can then be incorporated into an oligopeptide in solid-phase peptide synthesis (SPPS). Additionally, *N*-methylation concurrently to peptide synthesis is also possible. Commonly used methods for SPPS of *N*-methylated peptides involve activating the amino group with 2-nitrobenzenesulfonamide and methylating agents, such as dimethyl sulphate [40–42]. Directly methylating peptides is challenging. Multiple amide bonds and primary amines of the amino acid chains are available for methylation, leading to poor site selectivity of any methylating reaction [39]. Therefore, either pre-methylated amino acids or short methylated peptides are incorporated into the full-length peptide. Direct *O*-methylation of an *O*-desmethyl coibamide A in solution has been demonstrated by Sable et al. [43]. The use of diazomethane in diethyl ether as methylating agent yielded the methylated synthetic coibamide A. All of the aforementioned methods are robust and well-established. However, they do have their drawbacks. The application of toxic agents such as MeI, TFA, or sodium cyanoborohydride can be harmful to both health and environment. Additionally, the need for constant protection and deprotection steps is time-consuming and results in low atomic economy, while also increasing the use of solvents, energy, and chemicals. Furthermore, side reaction such as racemisation, fragmentation, deletions, and double or triple methylations can lead to lower yields and hinder the coupling of further amino acids after *N*-methylation [44].

**Ribosomal synthesis in vitro**. A cell-free translation system termed “protein synthesis using recombinant elements” (PURE) was developed by Shimizu et al. [45]. PURE contains all the necessary components of the *Escherichia coli* ribosomal machinery, such as the ribosome, initiation, elongation, and release factors, and pre-charged tRNAs. In the first step, mRNA or a plasmid containing the desired sequence is introduced into the system. After transcription and translation, the final peptide is purified and can be used for downstream applications (Figure 2) [45–46]. The incorporation of methylated amino acids relies upon the supply of the correct tRNAs. In this case, the tRNAs are prepared in advance and are not recycled in the system. The amino acids are methylated either prior to tRNA binding or after. The efficiency of incorporating unnatural amino acids is lower, but Merryman and Green have demonstrated that an excess of tRNA can overcome this problem [47]. When attempting to incorporate both methylated and unmethylated versions of the same amino acid, simply supplying both tRNAs is not sufficient, as the ribosome has a higher selectivity for the tRNA charged with the natural amino acid. This can be addressed by synthesising tRNA for a stop codon charged with the desired modified amino acid. This approach is limited to the addition of two unnatural amino acids, as the third stop codon is needed for termination. Another option is the reassignment of sense codons and supplying the desired chemically charged tRNAs. This has been demonstrated to work for up to 12 unnatural amino acid analogues [48–52]. The PURE system is a well-established method for rapidly expressing functional proteins. However, synthesising peptides with unnatural amino acids remains a labour-intensive process. tRNAs charged with unnatural or modified amino acids cannot be easily replenished within the system and must be prepared beforehand. The incorporation of unnatural amino acids by the ribosome is less efficient and leads to incomplete translation, resulting in shunt products. Additionally, ribosome recycling efficiency and synthesis of full length proteins declines with length of the primary sequence; Li et al. demonstrated this for proteins of up to 224 kDa [35]. And even when full-length peptides are synthesised, they may not always be active due to the absence of chaperones that aid in the proper folding of the nascent peptide chain [35,44].

**Enzymatic synthesis**. Another option is enzymatic peptide methylation. Biocatalysis provides an efficient and sustainable alternative to chemical synthesis strategies. Both NRPS and RiPP biosynthetic machineries can be used for cell-based or in vitro strategies [53]. Especially well suited as biocatalysts are MTs from RiPP pathways, as they demonstrate high substrate flexibility.

**Figure 3:**
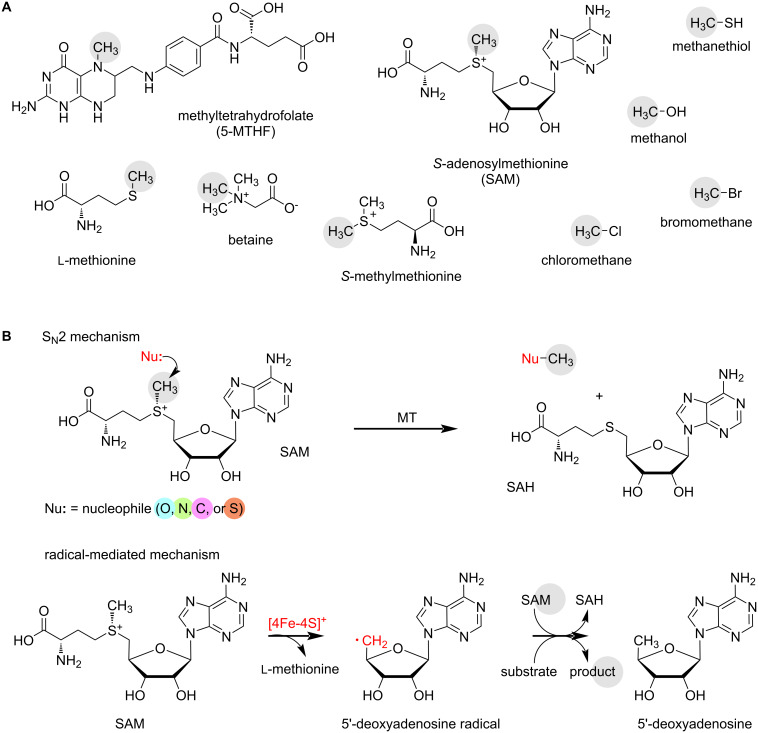
Biological methylation. A) Methyl donors from biological systems. The transferred methyl group is highlighted in grey. B) General reaction schemes of biological methylation reactions.

SAM is the universal methyl donor of biological methylation reactions, and it is the main outcome of the methionine cycle. Following transfer of the methyl group, SAH is formed, which is then hydrolysed to ʟ-homocysteine and adenosine by an SAH hydrolase. ʟ-Homocysteine is remethylated to ʟ-methionine by the enzyme methionine synthase, which utilises 5-methyltetrahydrolate (5-MTHF) as a methyl donor. 5-MTHF is a constituent of the folate cycle, which encompasses the intermediates tetrahydrofolate (THF) and 5,10-methylene-THF (5,10-CH_2_-THF). 5,10-CH_2_-THF is formed from THF and ʟ-serine, which provides the methyl group in this case. Another methyl donor that provides a C1 building block for ʟ-methionine and subsequent SAM synthesis is betaine. Betaine-homocysteine MT remethylates ʟ-homocysteine to ʟ-methionine. Non-SAM-dependent MTs catalyse B_12_-dependent methylations using the cofactors adenosylcobalamin (AdoCbl) and methylcobalamin (MeCbl). In such biological processes, MTHF can serve as a methyl donor, but also smaller molecules such as methanol and methanethiol [54–55]. Further low-molecular methyl donors are known from biological systems, including chloromethane (CH_3_Cl), bromomethane (CH_3_Br) [56], and *S*-methylmethionine [57] (Figure 3).

As mentioned above, RiPP MTs use SAM as a methyl donor in their enzymatic methylation reactions. The following section provides an overview of various RiPP maturases that methylate different functional groups.

### Methyltransferases from RiPP pathways

Methyltransferases can be classified based on various factors, such as their substrates (small molecule MTs, protein MTs, or RNA/DNA MTs), the atom that accepts the methyl group (oxygen = *O*-MTs, nitrogen = *N*-MTs, carbon = *C*-MTs, sulphur = *S*-MTs, or halide = H-MTs), metal or cofactor dependence (Mg^2+^-dependent, metal-independent, cobalamin-dependent), common structural folds (class I–V, with class I being the largest group, characterised by the Rossmann fold) [58], or catalytic mechanism (S_N_2 mechanism, radical mechanism, Figure 3) [59]. This review categorises RiPP MTs based on the acceptor atom, describing *O*-, *N*-, *C*-, and *S*-MTs; halide MTs have not (yet) been identified in RiPP pathways. The enzymes described below are either conventional SAM-dependent MTs or radical SAM (rSAM) MTs; rSAM MTs are one subfamily of the large rSAM enzyme superfamily, which encompasses enzymes catalysing a broad range of reactions [60]. There are different rSAM MT classes, description of the classes follows below in the *C*-MT section. Unlike conventional MTs, which follow an S_N_2 mechanism, rSAM MTs can methylate non-activated carbons.

#### *O*-Methyltransferases

The predominant group of MTs are *O*-MTs, which perform oxygen-directed methylation [59]. A range of natural products are known to undergo *O*-methylation [61], improving membrane permeability, absorption, and oral bioavailability [61–62]. The most extensively studied *O*-MTs are those that methylate catecholic hydroxy groups [63]. Several *O*-methylation modifications have been observed during the maturation of different RiPPs.

One example is the biosynthesis of thiopeptide GE2270, which is produced by the actinomycete *Planobispora rosea* ATCC53733 as a complex of related metabolites that differ in the number of methyl groups installed on the macrocyclic core. The *pbt* cluster, responsible for the synthesis of GE2270, encodes four MTs. Among these, only PbtM4 is an *O*-MT. It methylates thiazole D, resulting in a *C*-methylene-*O*-methyl-thiazole. The other MTs of the *pbt* cluster, one *N*-MT (PbtM1) and two *C*-MTs (PbtM2 and PbtM3) [64], will be discussed in later sections.

**Figure 4:**
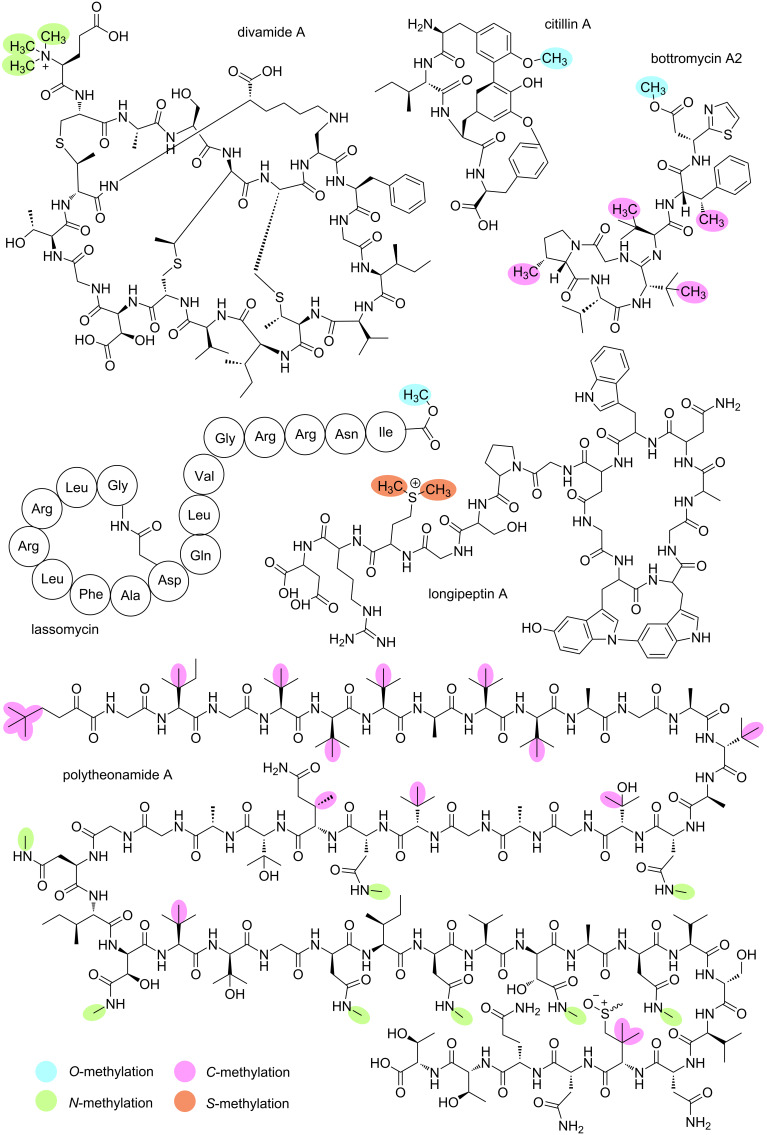
Chemical structures of RiPPs with diverse *O*-, *N*-, *C*-, and *S*-methylations. Amino acids of lassomycin are shown in the three-letter code. For longipeptin A, the stereochemistry has not been fully determined yet, one of two proposed positional isomers of the biaryl link between Trp2 and Trp3 is depicted [66].

*Myxococcus xanthus* DK1622 and other strains of *M. xanthus* produce the peptide cittilin A (Figure 4). The novel RiPP class of cittilins was established based on its unique bicyclic structure. Experimental evidence suggests that *O-*methylation occurs in the last biosynthetic step by the MT CitC [65]. Further *O*-MTs can be classified based on a common acceptor group of modification, namely C-terminal carboxy-MTs and peptide/protein ʟ-aspartyl *O*-MTs (PAMTs).

#### C-terminal carboxymethyltransferases

Carboxy-MTs catalyse the formation of a methyl ester by methylating the oxygen atom within the hydroxy group of a carboxylic acid. SAM is used as a donor of the methyl group [67–69]. The linear cyanobactin aeruginosamide B, produced by *Microcystis aeruginosa* PCC 9432, was the first RiPP reported to be *O*-methylated at its C-terminus [70].

**Figure 5:**
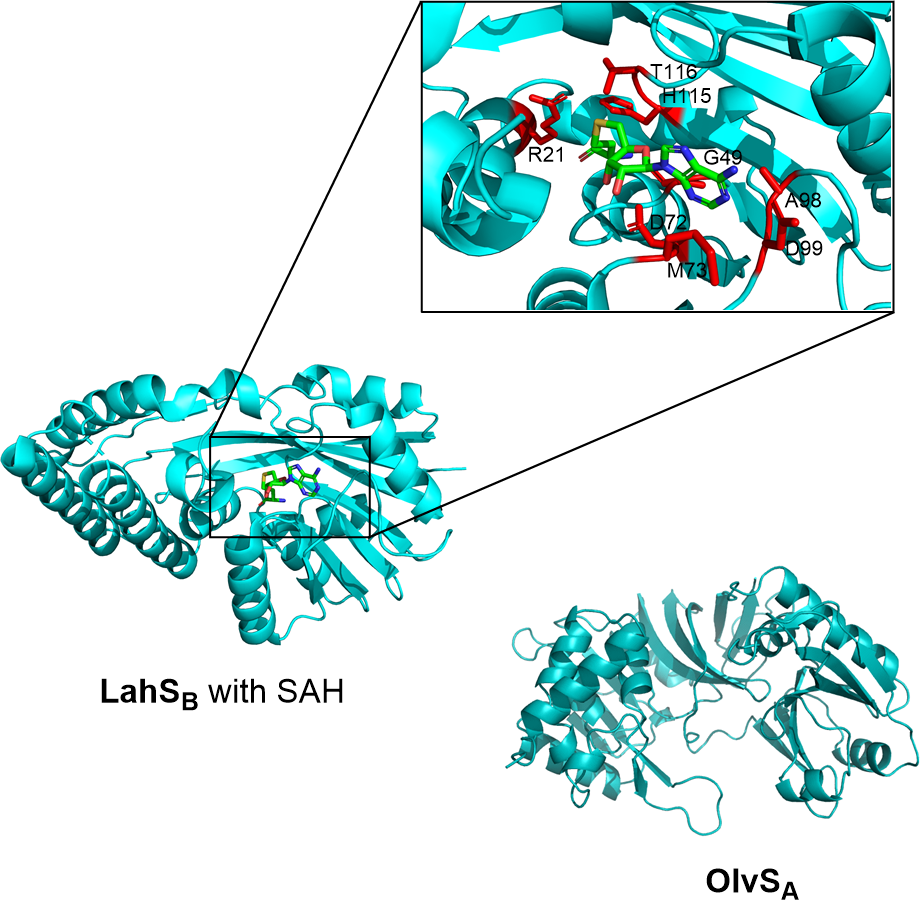
The three-dimensional structures of the conventional *O*-MTs OlvS_A_ (model structure calculated by Colabfold [75] and visualised by PyMOL [76]) and LahS_B_ with bound SAH, PDB ID: 6UAK (https://doi.org/10.2210/pdb6UAK/pdb), [72]. The residues involved in SAH binding are depicted in red.

LahS_B_ is a SAM-dependent MT encoded in the *lah* BGC of *Lachnospiraceae bacterium* C6A11. It belongs structurally to class I MTs and was the first MT observed acting on the C-terminus of lanthipeptides. Furthermore, it is the second occurrence of an *O*-MT acting on lanthipeptides, alongside the LanS_A_-type *O*-MTs [71]. LahS_B_ methylates four out of seven LahA precursor peptides at the C-terminal carboxy group of different amino acids, namely methionine, isoleucine, and valine. This demonstrates its tolerance to various substrates. LahS_B_ does not require the leader peptide or other modifying enzymes to carry out its function [72], making it a promising tool for biocatalytic applications. The co-crystallisation of LahS_B_ with bound SAH provides important details about the structure–function relationship, the substrate–enzyme interaction, and the cofactor binding site (Figure 5). This structural information, including the residues involved in binding, is essential for enzyme engineering and rational design [73–74].

*Lentzea kentuckyensis* sp. produces lassomycin (Figure 4), a lasso peptide, with a methyl ester at the C-terminus. The *las* cluster encodes LasF, which is predicted to function as an *O*-MT. Since no other putative MT is encoded in the *las* cluster, LasF is proposed to methylate lassomycin [77].

#### PAMTs – peptide/protein ʟ-aspartyl *O*-methyltransferases

**Figure 6:**
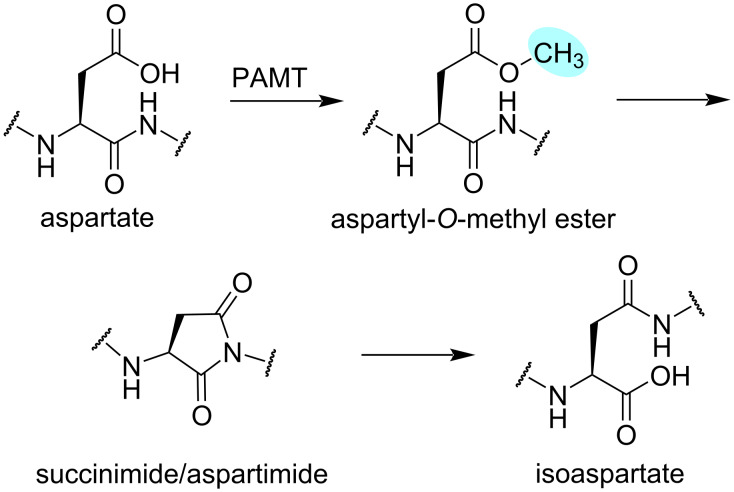
Reaction scheme of the PAMT´s catalysis, leading to the enzymatic conversion of aspartate to aspartyl-*O*-methyl ester, followed by a two-step non-enzymatic conversion via succinimide/aspartimide to isoaspartate. The methyl group is highlighted in blue. The reverse reaction with isoaspartate as substrate of PAMTs is also known.

PAMTs share a conserved C-terminal domain, substantial sequence homology, and a common enzymatic activity. They catalyse the conversion between aspartate and isoaspartate with aspartyl-*O*-methyl ester and aspartimide intermediates (Figure 6). The activity of most PAMTs depends on a cyclised precursor peptide. Modification of the aspartate/isoaspartate residue was found only in a hairpin-like or the macrocyclic region [71,78]. The underlying purpose of this structural requirement of PAMTs has not yet been demonstrated.

OlvS_A_, an *O*-MT encoded in the *olv* cluster from *Streptomyces olivaceus* NRRL B-3009 (Figure 5), is involved in the biosynthesis of the class I lanthipeptide OlvA(BCS_A_). It catalyses the conversion of a highly conserved ʟ-aspartate to ʟ-isoaspartate by methylation of a side chain carboxylate group of ʟ-aspartate, resulting in an ʟ-aspartyl-*O*-methyl ester. The ʟ-succinimide intermediate that is formed is non-enzymatically hydrolysed to the final isoaspartate. Experimental proof demonstrated that OlvS_A_ operates without the need for leader peptide recognition; instead, it relies on an already cyclised core peptide to exert its effects on the precursor peptide. Furthermore, SAM was shown to be an obligate co-substrate for OlvS_A_ [71]. Crystallographic data of OlvS_A_ is not yet available. The predicted three-dimensional structure displays substantial differences from the crystal structure of LahS_B_ [RMSD: 8.152 Å (109 to 109 atoms)], demonstrating the structural diversity among *O*-MTs (Figure 5).

*Streptomyces bottropensis* produces various bottromycins, including bottromycin A2 (Figure 4) [79]. The precursor peptide consists of an N-terminal core and a rare C-terminal follower region. The biosynthesis of bottromycin involves several methylations, catalysed by four different MTs encoded in the BGC. Gomez-Escribano et al. named the BGC in which the MTs are encoded *bmb* and proposed their putative function [80]. Huo et al. named the same BGC *bot* and experimentally determined the function of the MTs. BmbA/BotOMT is the only *O*-MT involved in the biosynthesis of bottromycin [81]. It *O*-methylates the aspartate by transforming its carboxylic acid group to a methyl ester in the ultimate step in biosynthesis [79]. The remaining methylations are catalysed by *C*-MTs, which will be addressed in a following section.

### *N*-Methyltransferases

*N*-Methylations of peptides offer several advantages for peptide therapeutics, including conformational modulation, influence on receptor subtype specificity, increased proteolytic resistance, and improved oral bioavailability and cell permeability [82–83]. *N*-Methylation can occur at the N-terminal amide, the peptide backbone, as well as on amino acid side chain amides (Figure 1). While traditional synthesis of *N*-methylated peptides is challenging and time consuming, installation via RiPP maturases provides an easier, quicker, and greener approach to confer these attributes to different peptide natural products. Although certain *N*-methylations may affect selectivity and bioactivity, a study on somatostatin derivatives showed that *N*-methylating the backbone improved oral bioavailability without affecting biological activity and selectivity [84].

#### α-*N*-Methyltransferases/*N*-terminal methyltransferases

The α-amino group of N-terminal amino acid residues can either be mono-, di-, or trimethylated. This methylation step occurs after the core peptide is cleaved from the leader and can enhance the metabolic stability of the final peptide natural product [85].

Crocagin A is the first RiPP to be isolated from Myxobacteria. It was specifically isolated from *Chondromyces crocatus* Cm C5. The core peptide is composed of only three amino acids: isoleucine, tyrosine, and tryptophan. It forms a tetracyclic core system that contains a tetrahydropyrroloindoline, which is unprecedented in bacterial natural products. Crocagin biosynthesis starts with formation of the central ring structure, followed by cleavage of the core peptide by peptidase CgnD. The final steps involve *N*-methylation by CgnL and carbamoylation by CgnI. It is unclear which modification is installed first. CgnL is a class I SAM-dependent MT. The closest structural homologues with defined biosynthetic pathways are CcbJ and KedS8. These enzymes *N*-methylate the antibiotic lincosamide celesticetin and the anticancer protein kedaricidin, respectively. Crocagin A is a potent inhibitor of the RNA binding protein CsrA, which is a promising target for new anti-infective compounds. However, drug development is hampered by poor cellular uptake [86–87].

In the biosynthesis of cypemycin in *Streptomyces* sp. OH-4156, CypM adds two methyl groups to the N-terminal alanine residue. Cypemycin has a very narrow activity spectrum and loses all activity when unmethylated. Although the mode of action is unclear, the methylations are essential to the bioactivity. CypM methylates many oligopeptides that mimic the cypemycin N-terminal sequence, and unrelated peptide antibiotics. Additionally, CypM methylates short oligopeptides with N-terminal alanine, serine, threonine, glycine, and methionine residues, as well as the antibiotics nisin and haloduracin with good efficiency. Nisin was methylated on its N-terminal isoleucine and on the side chain of Lys12, where two methylations occurred. It is important to note that N-terminally methylated nisin retains its antibiotic activity, with MIC (minimal inhibitory concentration) values of 1 µM and 4 µM against *Lactococcus lactis* and *Bacillus subtilis*, respectively. Haloduracin is a two-component lanthibiotic that is methylated at both cyclised N-termini by CypM. These experiments demonstrate that CypM has a relaxed substrate specificity, which makes it interesting for bioengineering applications [88].

Other examples of terminal *N*-methylation can be found in the dikaritins, a family of fungal RiPPs. Among the first members of this family to be discovered were the ustiloxins. Ustiloxins were first identified in 1994, but their BGC was not identified until 2013 [89–90]. The biosynthesis of ustiloxin starts with the UstA precursor, which contains a 16-fold repeat of the Tyr-Ala-Ile-Gly core. Following the cleavage of the precursor into tetrapeptides, the tetrapeptides are cyclised via the tyrosine and isoleucine side chains, and then *N*-methylation is installed by UstM on the N-terminal tyrosine residue [91–92]. Another member of the dikaritin family is phomopsin. All phomopsins contain at least one *N*-methylated tyrosine. In vivo studies have attributed the MT activity to PhonM, and in vitro studies have also demonstrated MT activity on phomopsin A, resulting in the dimethylated phomopsin E [93].

In the biosynthesis of plantazolicin, PznL (BamL) catalyses a reaction that is highly analogous to that of PhonM, involving the α-*N*-dimethylation of arginine. Plantazolicin was isolated from *Bacillus amyloliquefaciens* FZB42 and it mainly exhibits bactericidal activity against *Bacillus anthracis* [94–96]. The dimethylation is essential for its bioactivity; desmethyl plantazolicin showed MIC values >128 µg/mL for *B. anthracis*, while the dimethyl species showed MIC values between 2–4 µg/mL [96]. In contrast to CypM, PznL has higher substrate specificity towards its peptide substrate. It required more than just an N-terminal arginine as a recognition element. PznL cannot accept peptide substrates that lack the polyheterocyclic peptide backbone modifications of plantazolicin [94].

Divamide A (Figure 4) was first isolated from a marine tunicate, *Didemnum molle*, extract, based on its anti-HIV activity in a bioassay-guided fractionation. Further investigation on the identification of the BGC revealed that the tunicate symbiont, *Prochloron didemni*, is actually responsible for producing divamide A. Divamide A contains a trimethylated glutamate at the N-terminus (Figure 4), which is installed by DivMT as the final step in divamide A biosynthesis. The presence of the cyclic lysinoalanine moiety is required for this post-translational modification. The addition of other N*-*terminal amino acids is not tolerated by DivMT. It appears that the MT has a high specificity for the overall scaffold of divamide A [97].

#### Backbone-*N*-methyltransferases

**Figure 7:**
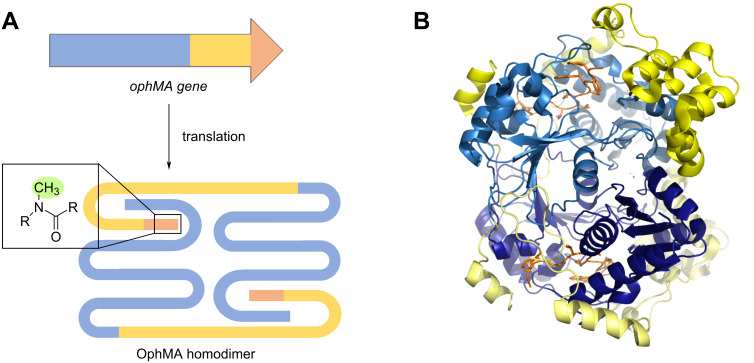
Structural organisation of the OphMA homodimer. A) Schematic representation. The MT domain is coloured in blue; the leader peptide is coloured in yellow, and the core peptide is coloured in orange. Methylation of the backbone is highlighted in green. B) Three-dimensional structure of the OphMA homodimer, PDB ID: 6TCS (https://doi.org/10.2210/pdb6tsc/pdb) [99]*.*

Borosins are a particular interesting RiPP family, and the first family containing maturases capable of backbone-*N*-methylation. Omphalotin A is the founding member of this RiPP class. It is biosynthesised by *Omphalotus olearius* and has nematocidal properties. In types I through III of borosins, the precursor peptide is fused to the MT domain; OphMA designates the fused precursor-MT from the omphalotin A BGC (Figure 7). After auto-modifying the core, the precursor is cleaved and the MT domain is presumably degraded without catalysing any further methylating reactions [98]. The OphMA fusion functions as a homodimer, with the active site of the interlocking dimers modifying the substrate peptide (Figure 7) [99]. The MT domain of OphMA is especially interesting due to its relaxed substrate specificity. In experiments where the precursor was replaced with unrelated peptide natural products amino acid sequences, such as cyclosporin and dictyonamide, the MT domain methylated the backbone up to 5 times, but the pattern did not correspond to that of the natural modified core peptide of the precursor domain. A subsequent study demonstrated that the MT domain can also *N*-methylate the amide bonds of non-natural amino acids fused to the already folded MT domain of OphMA. These results show that the MT domain of OphMA is highly permissive in terms of core peptide sequences [100–101].

**Figure 8:**
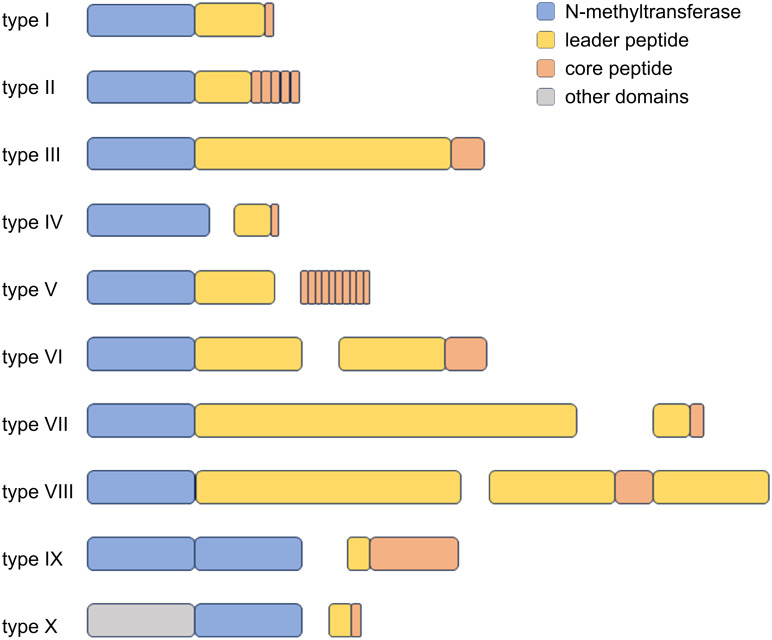
Overview of the protein architectures and core peptide compositions of borosin *N*-MTs as defined by Imani et al. Types I–III are of fungal origin, types IV–X are of bacterial origin [103].

Type IV borosins are more distantly related homologues found in bacteria; although it was originally believed that borosins occur mainly in fungi, Cho et al. and Imani et al. both demonstrated that type IV borosin BGCs are widespread in bacteria and have additionally defined classes V through X (Figure 8) [102–103]. The type IV MT SonM was first described and characterised by Miller et al. using *Shewanella oneidensis* MR-1 as a model system [104]. While the MT still acts as a homodimer, the precursor SonA bears a borosin binding domain, which allows SonM-SonA dimers to arrange into a heterotetramer for methylation of the backbone. SonM installs two methylations on the peptide backbone of SonA at Leu63 and Ile65. The borosin binding domain could potentially be exploited for engineering of other precursors and synthesising of novel peptides [104]. It was demonstrated that SahM from *Shewanella* sp. HN-41 has a substrate flexibility similar to that of the MT domain of OphMA, thus expanding the scope of type IV borosins. SahM was able to methylate different alanine-substituted precursors, as well as completely unnatural core sequences of OphMA and cyclosporin A comparable to the native precursor.

Over 50 putative borosin MTs have been identified in a study by Quijano et al. [105]. Heterologous expression of 11 precursor–MT pairs highlighted different methylation patterns. All of the MTs tested appear to methylate in an N-to-C-terminal direction on primarily hydrophobic core peptides [105]. If other MTs display similarly lax specificity to their core peptides as the MT domain of OphMA and SahM, a varied toolbox of MTs could be envisioned for use in the synthesis of peptide therapeutics [102].

EreM is a backbone-MT which does not belong to the borosin family. It is part of the pythonamide biosynthesis in *Candidatus* Eudoremicrobium spp. Pythonamide belongs to the proteusin RiPP family, it has a 46-amino acid core peptide on which up to six *N*-methylations are installed. EreM is an FkbM-like MT and catalyses this reaction on different valine residues of the core peptide. While the first methylation can be installed independently of other modifications, the other five are only installed when EreM is co-expressed with the oxygenase EreI [106].

#### *N*-Methylation on amino acid side chains

The polytheonamides are heavily modified peptides that belong to the proteusin family of RiPPs. They are produced by the tunicate symbiont *Candidatus* Entotheonella factor and exhibit membrane activity against Gram-positive bacteria and are highly cytotoxic. Cytotoxicity was tested with murine leukaemia cells; polytheonamide A, B, and C showed IC_50_ values between 68 and 78 pg/mL [107–109]. While originally thought to be of NRPS origin due to their extensive modifications, Freeman et al. discovered in 2012 that polytheonamides are actually of ribosomal origin [108]. Polytheonamides A and B contain eight methylated asparagine residues (Figure 4), all of which are installed by the MT PoyE. Interestingly, two of these methylated asparagine residues also carry a hydroxy group at the β-carbon, which does not appear to affect the methylations. Renevey and Riniker demonstrated that the *N*-methylated asparagine residues of polytheonamide B are essential for the stability of its β-helical structure; reversion of the *N*-methylation led to complete conformation loss. The methylated asparagine residues at positions 16, 22, and 28 form an intramolecular chain of hydrogen bonds along the outside of the β-helix. Hydrogen atoms from the amide methyl group form hydrogen bonds with the oxygen of the neighbouring *N*-methylated asparagine amino acid side chain. This chain of hydrogen bonds stabilises the β-helical structure of polytheonamide B [110]. PoyE acts independently of the dehydratase and epimerase activities of other maturases in the cluster, but the MT is more efficient on ᴅ-asparagine compared to ʟ-asparagine. PoyE exhibits a degree of tolerance towards precursor variations. The insertion of a GNINVN motif into the precursor, which represents one turn of the helical structure, did not affect the activity of PoyE: this elongated precursor was methylated by PoyE with comparable efficiency to the natural precursor. However, simple swapping of amino acids on the precursor sequence yielded mixed results. These findings suggest that PoyE has a plastic yet regiospecific activity that could potentially be exploited in bioengineering applications [111].

Aeronamide A is another member of the channel-forming, cytotoxic proteusins. It is biosynthesised by *Microvirgula aerodenitrificans*. Like polytheonamide B, aeronamide A shows cytotoxic activity against HeLa cells, with an IC_50_ of 1.48 nM. AerE, the PoyE homologue, installs five methylations on the amides of Asn25, Asn31, Asn37, Asn41, and Asn43 (putative localisation) [112]. The activity of AerE is independent of the leader peptide; nevertheless, it only methylates ᴅ-asparagine residues. Additionally, AerE is independent of secondary structure, in co-crystallisation experiments the precursor is bound in an extended, unstructured configuration to AerE [113]. Otherwise, the biosynthetic machinery of aeronamide A seems to be permissive to other core sequences. Swapping the core sequence with other proteusin core structures, such as the polytheonamides core and a *Rhodospirillaceae* sp. proteusin precursor, demonstrated a relaxed substrate specificity for all maturases in the *aer* BGC: All hybrid precursors were extensively modified with dehydrations, *C*-methylation, *N***-**methylations, and epimerisations comparable to the AerA precursor [112–113].

PbtM1 from the thiopeptide GE2270 BGC is another example of a side chain amide-*N-*MT [114]. PbtM1 methylates Asn4 in the GE2270 precursor peptide. Substrate specificity appears to be pronounced, as amino acid substitutions in the core were not tolerated by the biosynthetic machinery. PtbM1 acts independently of other modifications since linear intermediates after enzyme deletion almost always show Asn4 methylation. Further investigation into the substrate scope of PbtM1 would be interesting to determine its potential in peptide engineering [64].

An example for lysine *N-*methylation can be found in epichloëcyclins. This family of RiPPs is found in endophytic fungi of the genus *Epichloë*. Epichloëcyclins A–E are cyclic heptameric peptides, all produced from the same precursor GigA. They carry a dimethylated lysine residue; this modification is installed as a final step in the biosynthesis by GigC. Genome mining showed that analogues of the GigA precursor are widespread in fungal genomes, exploration of these BGCs may lead to the discovery of further *N*-MTs [115–116].

### *C*-Methyltransferases

*C*-MTs are less prevalent than *O*- or *N*-directed MTs; they transfer a methyl group to a carbon atom [117]. *C*-Methylation of drugs can enhance their potency enormously [14]. Several *C*-MTs are involved in RiPP maturation, all *C*-MTs described in the following section are classified as rSAM *C*-MTs.

#### Radical SAM *C*-methyltransferases

**Figure 9:**
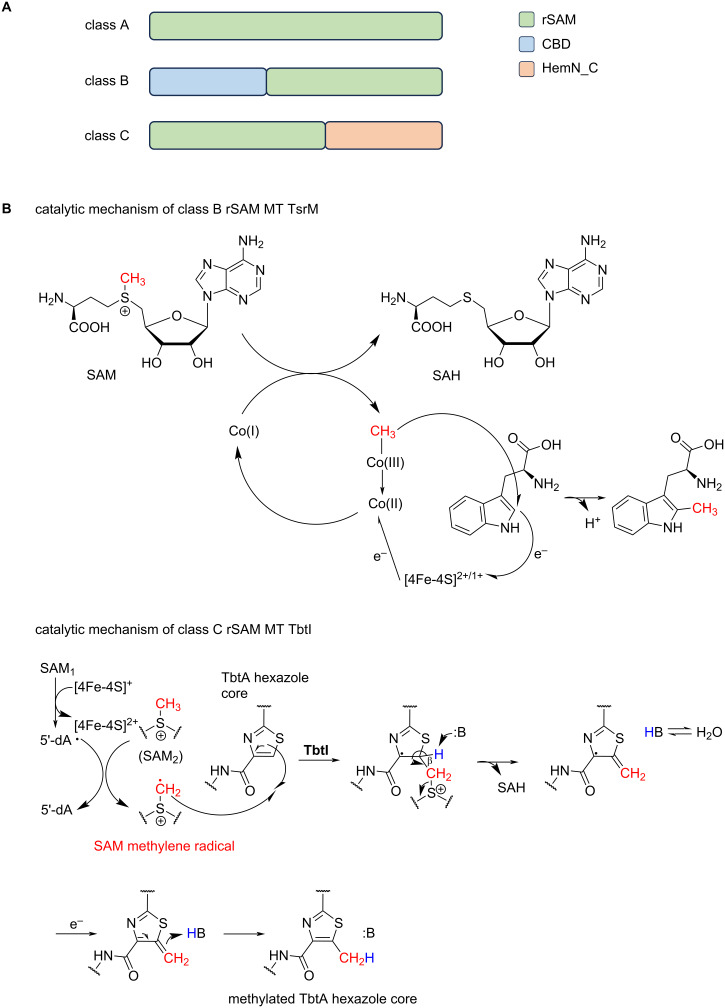
Radical SAM *C*-methyltransferases. A) The different rSAM MT classes containing different functional domains. Class B includes a cobalamin binding domain (CBD) and class C a domain similar to HemN_C, which is an oxygen-independent coproporphyrinogen-III oxidase (CPO). B) Catalytic mechanisms of rSAM-mediated methylation are depicted exemplary for class B (TsrM) and class C (TbtI) RiPP MTs.

Radical SAM enzymes typically contain a conserved CxxxCxxC motif. The cysteine residues of this motif coordinate a [4Fe-4S] cluster. rSAM MTs can be classified based on their domain structure. Class A rSAM MTs hold solely an rSAM domain, while classes B and C each contain an additional functional domain [118]. The activity of class B rSAM MTs is B_12_-/cobalamin-dependent. The proposed structure of B_12_/rSAM-MTs consists of a didomain protein consisting of a cobalamin binding domain (CBD) at the N-terminus and an rSAM domain at the C-terminus (Figure 9) [119].

In addition to its different enzyme structure, a distinct mechanism of methylation than the classical S_N_2 mechanism is employed. Reductive cleavage of SAM generates a 5'-deoxyadenosyl (dAdo) radical, which putatively produces a radical intermediate by abstracting a hydrogen atom from the peptide substrate. Methylation occurs by reaction of methylcob(III)alamin (MeCbl) with the substrate. Then, cob(II)alamin is released, reduced to cob(I)alamin, and methylated by a second SAM molecule to restore MeCbl [118,120–122].

The biosynthesis of bottromycin involves various *C*-methylations catalysed by three different enzymes, in addition to the *O*-methylation mentioned in a previous section. Specifically, phenylalanine is methylated by BmbB/BotRMT1, two of the three valines present are methylated by BmbF/BotRMT2, and proline is methylated by BmbJ/BotRMT3 (Figure 4) [81]. Three derivatives of bottromycin were obtained through fermentation of the natural producer. The proline residue methylation pattern varies, with bottromycin A2 being monomethylated, bottromycin B2 being unmethylated, and bottromycin C2 being dimethylated [81,123].

**Figure 10:**
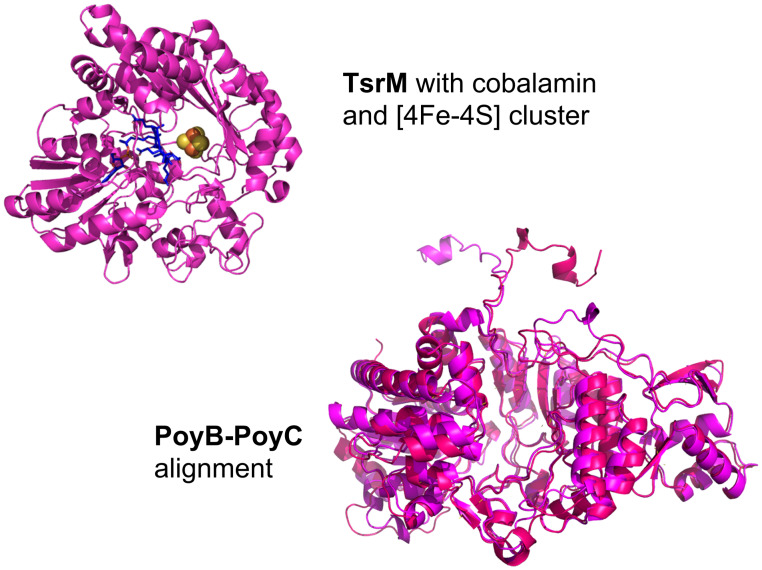
The three-dimensional structures of the rSAM *C*-MTs TsrM with bound cobalamin and [4Fe-4S] cluster (PDB ID: 6WTE (https://doi.org/10.2210/pdb6WTE/pdb), [129]) and the structural alignment of PoyB and PoyC (model structures calculated by Colabfold [75] and visualised by PyMOL [76]). The RMSD of TsrM–PoyB is 4.032 Å (214 to 214 atoms), of TsrM–PoyC is 7.067 Å (329 to 329 atoms).

Polytheonamides are originally derived from the marine sponge *Theonella swinhoei*, which is colonised by various symbiotic bacteria. The polytheonamide encoding BGC *poy* includes two B_12_-dependent rSAM *C*-MTs, namely PoyB and PoyC. In silico modelling and alignment of the structures of PoyB and PoyC indicate their high structural similarity with a RMSD of 0.907 Å (552 to 552 atoms) and a sequence similarity of 64.56% (Figure 10). PoyC is the first enzyme in the class B rSAM MTs to perform C_β_-methylations, and exclusive methylates ʟ-configured amino acids. Furthermore, PoyC contains a unique Cx7Cx2C rSAM motif. In the polytheonamide biosynthesis, PoyC methylates Ile3 and valine 5, 10, and 21. The activity of PoyC and PoyB is spatially separated, as PoyC only operates on residues 1 to 21, while PoyB operates only on residues 23 to 49. PoyB methylates Val31 and Met45, albeit with low efficiency. PoyB was proposed to produce the N-terminal *tert*-butyl group of the polytheonamide A (Figure 4), but later PoyC was demonstrated to be responsible for this modification [111,124]. This structural feature conveys hydrophobicity to the N-terminus and is in line with the decreasing hydrophobicity towards the C-terminus, named "axial amphipathicity". Thus, this methylation is thought to play an important role in correct target membrane insertion and the associated cytotoxic effect [125]. TsrM is another cob(II)alamin-dependent *C*-MT (Figure 10). This enzyme is involved in the biosynthesis of thiostrepton A, which is known to be produced by different *Streptomyces* strains [126–127]. During the maturation of thiostrepton A, a quinaldic moiety is formed from tryptophan. TsrM catalyses the initial step by transferring a methyl group from SAM to the electrophilic carbon atom C2 of tryptophan. In the process of biosynthesis, the quinaldic moiety is eventually integrated into the precursor peptide TsrA. Besides its unique methylation reaction, TsrM has a high turnover rate and shows a great substrate ambiguity [128]. TsrM was crystallised, revealing its structure, and enabling the localisation of bound cobalamin and the [4Fe-4S] cluster (Figure 10). The structural variation among *C*-MTs becomes apparent when comparing the crystal structure of TsrM with the predicted structures of PoyB and PoyC.

Class C rSAM MTs are enzymes with a HemN-like didomain structure, consisting of an rSAM core domain and a C-terminal domain similar to HemN. HemN is an oxygen-independent coproporphyrinogen-III oxidase (CPO) and is assigned as rSAM dependent [119,130]. The HemN-like domain is hypothesised to be involved in substrate binding. Most of the residues responsible for binding two SAM molecules in HemN are conserved in all class C rSAM MTs. Therefore, it is suggested that one SAM molecule is bound for the production of a dAdo radical, while a second SAM molecule serves as a donor of the methyl group. The precise mechanism of class C rSAM MTs remains elusive [118].

Previous sections already described the *O*- and *N*-MT encoded in the *pbt* cluster of *P. rosea*. The thiopeptide GE2270 encoded by *pbt* undergoes further regioselective modification through *C*-methylation of thiazoles. The two rSAM MTs PbtM2 and PbtM3 methylate thiazole E and thiazole D, respectively [64]. PbtM2 and PbtM3 show substantial sequence similarities to the *C*-MTs of other thiopeptides, such as Tbtl (biosynthesis of thiomuracin) and NosN (involved in the maturation of nosiheptide) [131–132].

Thiomuracin is produced by *Nonomuraea* sp. and encoded in the *tbt* cluster [132]. During maturation it is methylated by the rSAM *C*-MT Tbtl, forming a 5-methylthiazole through *C*-methylation on an unactivated sp^2^ carbon centre of thiazole, likely thiazole 4. The analysis of Tbtl´s activity revealed its predominant independence from the leader peptide. Only one residue, Asn3, in the precursor peptide was shown to be crucial for activity [133].

*Streptomyces actuosus* contains the BGC *nos*, which encodes nosiheptide. The protein NosN encoded in *nos*, is responsible for catalysing the *C*-methylation of the peptide at C4 of the indolyl moiety. A unique characteristic of NosN, which distinguishes it from other known rSAM MTs, is that is does not generate SAH as a byproduct. This observation suggests a novel mechanism for indole C4 methylation by NosN [131,134].

### *S*-Methyltransferases

*S*-MTs are rare in RiPP biosynthesis and in natural product synthesis in general. There are few examples, and in most cases, the characterisation of the MT does not go beyond deletion studies to establish function, such as thiol *S*-methylation by SchX in the biosynthesis of the thiopeptide Sch40832, if it is identified at all [135–136]. In this section two *S*-MTs will be discussed.

The first MT is involved in the biosynthesis of an unidentified metabolite in *Candidatus* Entotheonella factor. The MT PtyS is part of a proteusin BGC *pty* and catalyses the *S*-methylation of up to four out of five cysteine residues in the precursor PtyA. In heterologous co-expressions of PtyA and PtyS, methylations were localised via LC–MS analysis to Cys9 and Cys15, with Cys9 being the preferred target. In the heterologous expression system, *S*-methylation of the cysteine residues can co-occur with lanthionine formation. When PtyM and PtyS are co-expressed, only one methylation of cysteine and three dehydrations could be observed. This may be due to the installed modifications inhibiting each other or due to limitations of the expression system [137].

LopH is a second example of RiPP *S*-MTs, and it is part of the longipeptin A biosynthesis (Figure 3). LopH is predicted to be responsible for *S*-methylation of methionine, yielding a sulphonium moiety on Met13. While *S*-methylation was shown via LC–MS and NMR analysis of the natural product, no knockout studies were performed to identify which enzyme is responsible for the *S*-methylation. The authors propose LopH as the most likely enzyme, as it shares modest sequence and structural similarity with other MTs. This also hints at a possible new MT family with LopH as its founding member. Future experimental work will be necessary to unequivocally assign this function to LopH [66].

### Future prospects: biocatalytic potential of RiPP methyltransferases

The substrate scope of several of the RiPP MTs described above has been investigated, and some show promising potential for biocatalytic applications, accepting a wide range of different precursor substrates. In many cases, these substrate scope analyses are performed by co-expressing different precursor peptides with the MT – in such in vivo systems, SAM supply or SAM regeneration is not required. However, using SAM supply or regeneration systems may be useful if the peptide substrate cannot be synthesised ribosomally, e.g., if it contains non-proteinogenic amino acids that cannot be produced by post-translational modifications of RiPP pathways.

SAM supply or SAM regeneration systems typically synthesise SAM from ʟ-methionine and adenosine 5’-triphosphate (ATP) using a methionine adenosyltransferase. The MT then utilises SAM for the methylation reaction and the resulting SAH is degraded to adenine and *S*-ribosylhomocysteine, or to adenosine and ʟ-homocysteine [4,21,25]. To achieve complete SAM regeneration, adenine or adenosine can be recycled to ATP, and ʟ-homocysteine can be remethylated to ʟ-methionine [21,25]. The SAM regeneration system is not only applicable to conventional SAM-dependent MTs but also to rSAM MTs and other SAM-dependent enzymes, such as amino-propyltransferases [26]. Furthermore, alternative alkyl groups such as ethyl, propargyl, allyl, benzyl, and nitrobenzyl groups can be transferred [31,138–142]. Such alkylation strategies have been successfully applied to small molecules such as coumarin [141], anthranilate [21], and pyrazole [24], as well as larger acceptor molecules such as the polyketide rapamycin [31] or DNA [143].

The use of RiPP MTs with SAM supply or SAM regeneration systems has the potential to greatly increase chemical diversity of RiPP natural products. The addition of alternative alkyl groups onto peptide substrates could further expand the range of products. RiPP MTs exhibit excellent specificity in the late-stage alkylation of peptide natural products.

## Conclusion

RiPP pathways are a valuable source of novel MTs that enable methylations at various positions of peptide substrates. MTs from RiPP pathways can supplement the MTs currently used for biocatalytic methylation reactions. If RiPP MTs, like many other MTs, also accept SAM-derivatives with larger alkyl groups as substrates, they can transfer various groups such as ethyl, allyl, propyl, and benzyl moieties. In vitro systems can be used to install specific modifications in peptide structures. The remaining challenges include combining various post-translational modifications and developing a medium to high throughput screening approach to subject interesting peptide drug candidates to various MTs and perform SAR studies (structure–activity relationship). Overall, RiPP MTs will be a useful tool for drug development; it will be intriguing to observe what the future holds.

## Data Availability

Data sharing is not applicable as no new data was generated or analyzed in this study.
